# The role of the immune response in developing tuberculosis infection: from latent infection to active tuberculosis

**DOI:** 10.3389/ftubr.2024.1438406

**Published:** 2024-09-30

**Authors:** Igor Kudryavtsev, Anna Starshinova, Artem Rubinstein, Anastasia Kulpina, Hong Ling, Min Zhuang, Dmitry Kudlay

**Affiliations:** ^1^Research Department, Almazov National Medical Research Centre, St. Petersburg, Russia; ^2^Laboratory of Cellular Immunology, Institute of Experimental Medicine, St. Petersburg, Russia; ^3^Department of Mathematics and Computer Science, St-Petersburg State University, St. Petersburg, Russia; ^4^Department of Microbiology, Immunology, Harbin Medical University, Harbin, China; ^5^Department of Pharmacology, Institute of Pharmacy, I. M. Sechenov First Moscow State Medical University, Moscow, Russia; ^6^Laboratory of Personalized Medicine and Molecular Immunology, NRC Institute of Immunology FMBA of Russia, Moscow, Russia; ^7^Department of Pharmacognosy and Industrial Pharmacy, Faculty of Fundamental Medicine, Lomonosov Moscow State University, Moscow, Russia

**Keywords:** tuberculosis, immune response, latent tuberculosis, active tuberculosis, pathogenesis, dendritic cells, T cells

## Abstract

Despite advancements in modern medicine, tuberculosis continues to be one of the leading causes of death globally. Findings indicate that COVID-19 may trigger the activation of tuberculosis infection (TB), leading to its spread. Despite the development of new immunological diagnostic methods for latent tuberculosis infection (LTBI), it is still unclear how the infection transitions to an active TB state. The goal of the study is to provide insights into the progression of tuberculosis infection from a latent to an active state. This article presents recent research data focused on investigating the pathogenesis of LTBI, particularly the immune responses in the interaction between *Mycobacteria tuberculosis* (Mtb) and the host. It describes the mechanisms of T-cell immunity and cytokine activation, supporting the concept of type 1, type 2, and type 3 immune responses. According to the conducted studies, Th17 cells have a significant role in the development of type 3 antigen-specific responses. The cytokines IL-6 and IL-23 activate STAT3, which is necessary to trigger the expression of Th17. Future research on the role of Th17 cells and cytokines, particularly IL-6 and IL-21, may be beneficial in understanding the shift from LTBI to active TB.

## 1 Introduction

In the first quarter of the 21st century, tuberculosis continues to be a significant problem that mankind has not yet been able to solve. Despite the achievements of modern medicine, this infectious disease remains one of the leading causes of mortality globally. Consequently, the prevalence of tuberculosis infection can be considered as an indicator of the socio-economic well-being of a country, reflecting the level of development of health care systems, the quality of life of the population and the effectiveness of measures taken to combat this pandemic (1, 2).

The problem of tuberculosis infection was not solved before the pandemic of the new coronavirus infection, despite successes in reducing morbidity and mortality by 2019, and has become even more urgent with the global spread of SARS-CoV-2 (*Severe acute respiratory syndrome-related coronavirus 2*) virus (3–6).

Despite all measures taken, starting in 2010, the estimated number of new cases increased from 8.8 million to 10.4 million by 2016, but between 2017 and 2020, there was a decline in this figure to 10.0 million cases (including 0.3 million people with HIV infection) (2, 5). In 2021, according to the WHO Global Report (2022), the number of new TB cases increased to 6.4 million cases (6).

Since 2018, there has been a steady downward trend of up to 1.2 million deaths among tuberculosis patients not infected with HIV-infection (2). Nevertheless, according to the WHO experts, in the post-coronavirus period (after the COVID-19 (*Coronavirus Disease 2019*) pandemic), against the background of a subjective reduction in morbidity rates, the mortality rate from tuberculosis infection may increase by 15%. It is assumed that this increase may be due to excessive burden on health systems, insufficient epidemiological monitoring of tuberculosis, and decreased quality and coverage of population with using X-ray (7, 8).

Against the background of the COVID-19 pandemic, as expected according to WHO data, in 2021 the number of new TB cases decreased to 7.8 mi and mortality increased to 1.3 million (9), which is associated with the termination of developed programmers to detect TB infection. SARS-CoV-2 virus has been shown to affect a reduced immune response (10). The findings suggest the possibility of activation of tuberculosis infection, leading to the spread of infection after COVID-19. The development and introduction of new immunological methods of diagnostics of latent tuberculosis infection (LTBI) do not allow us to answer the question about the transition of the organism to the state of active TB (11, 12). *The aim of the study*: To provide insights into the pathogenesis of tuberculosis infection from latent tuberculosis infection to active tuberculosis.

## 2 Pathogenesis of active tuberculosis and latent tuberculosis infection according to experimental studies

Tuberculosis infection (TB) is characterized by great variability at different stages of infection, from pre-malignant to manifest disease, depending on the virulence and quantity of the pathogen ingested, the duration of contact and routes of infection, and the state of host immunity. These characteristics do not allow the creation of an accurate animal model of the disease with the ability to replicate the development of the disease in humans (13).

The first host-pathogen interaction in tuberculosis occurs when *M. tuberculosis* is recognized by cells of the innate immune system (11). It is thought that at this point innate immunity is crucial for triggering the early antimycobacterial response, but this initial contact also appears to determine the progression of infection and the long-term control of *M. tuberculosis* spread. It is this primary interaction between pathogen and host cells that is a prerequisite for antigen capture and presentation by cells of the adaptive immunity system, as well as being responsible for subsequent responses aimed at regulating inflammation (12). However, cells of innate immunity are often ‘niches' for mycobacterial replication, and *M. tuberculosis* utilizes various strategies that reduce the efficiency of primarily innate immune system reactions in order to induce chronic infection (13).

Various models of pulmonary and extrapulmonary TB have been tested on laboratory animals considered most susceptible to TB infection, such as guinea pigs, rabbits and non-human primates, as well as on less susceptible animals, such as dogs and cats. Animal models of TB are used to study pathomorphological changes in organs and tissues, as well as to test the efficacy of new vaccines and methods of TB treatment and for other purposes. At the same time, different lines of animals are used to solve specific tasks, as well as methods of their infection (13, 14).

In a mouse experiment, it has been shown that lymph node involvement occurs within the first month after TB infection. *M. tuberculosis* is thought to be defined in macrophages, although infected dendritic cells (DCs) have been observed. Following aerosolized infection, *M. tuberculosis* are phagocytosed by alveolar macrophages, myeloid DCs, and neutrophils in the lungs (15, 16).

It has been shown that TB phagocytic cells of different phenotypes, with the predominant population of infected cells changing over time, and that myeloid DCs in the lungs and lymph nodes are the major population of cells infected by *M. tuberculosis*. It was also found that bacteria in the lymph node draining the lungs are transported there from the lungs by a CCL19/CCL21-dependent mechanism and that bacterial transport to the lymph node is a transient phenomenon despite chronic infection (17–19).

Lung cell population that are infected with *M. tuberculosis* with high frequency are relatively ineffective in stimulating Ag-specific CD4+ T lymphocytes, which may inhibit Ag presentation by MHC class II without decreasing in surface expression of MHC class II. These results suggest that Mtb targets DCs migration and Ag presentation *in vivo*, promoting persistent infection. While other respiratory viral and bacterial pathogens induce migration of DCs to lymph nodes to activate the immune system within 1-3 days after infection (8), this important process is delayed in TB (20, 21, 23).

Several studies have shown that Mtb-infected DCs do not migrate to the lymph node and present T cells until 9-11 days after infection with the tuberculosis pathogen. This delayed spread of Mtb into lymph nodes is thought by some authors to play a role in the increased susceptibility of C3H/HeJ mice to Mtb, compared to C57BL/6 mice (10, 11). DCs migration was also shown to be transient, slowing down after peaking at 21 days post infection, an interesting observation given the chronic nature of TB.

Not only migration functions of DCs but also of interstitial macrophages, which transport *M. tuberculosis* to lymph nodes, are dysregulated and relatively poorly stimulated T-cell responses to *M. tuberculosis* antigens (6, 12, 13).

One of the earliest (5-15 days after infection) observations in the lungs is inflammation of pulmonary lymphatic vessels (8). Marked enlargement of intrathoracic lymph nodes was determined about 20 days after infection, which progressed to severe lymphadenopathy 30 days infected by *M. tuberculosis* (14, 15). Lymph node involvement has also been reported in rabbit models (16).

**Figure 1:**
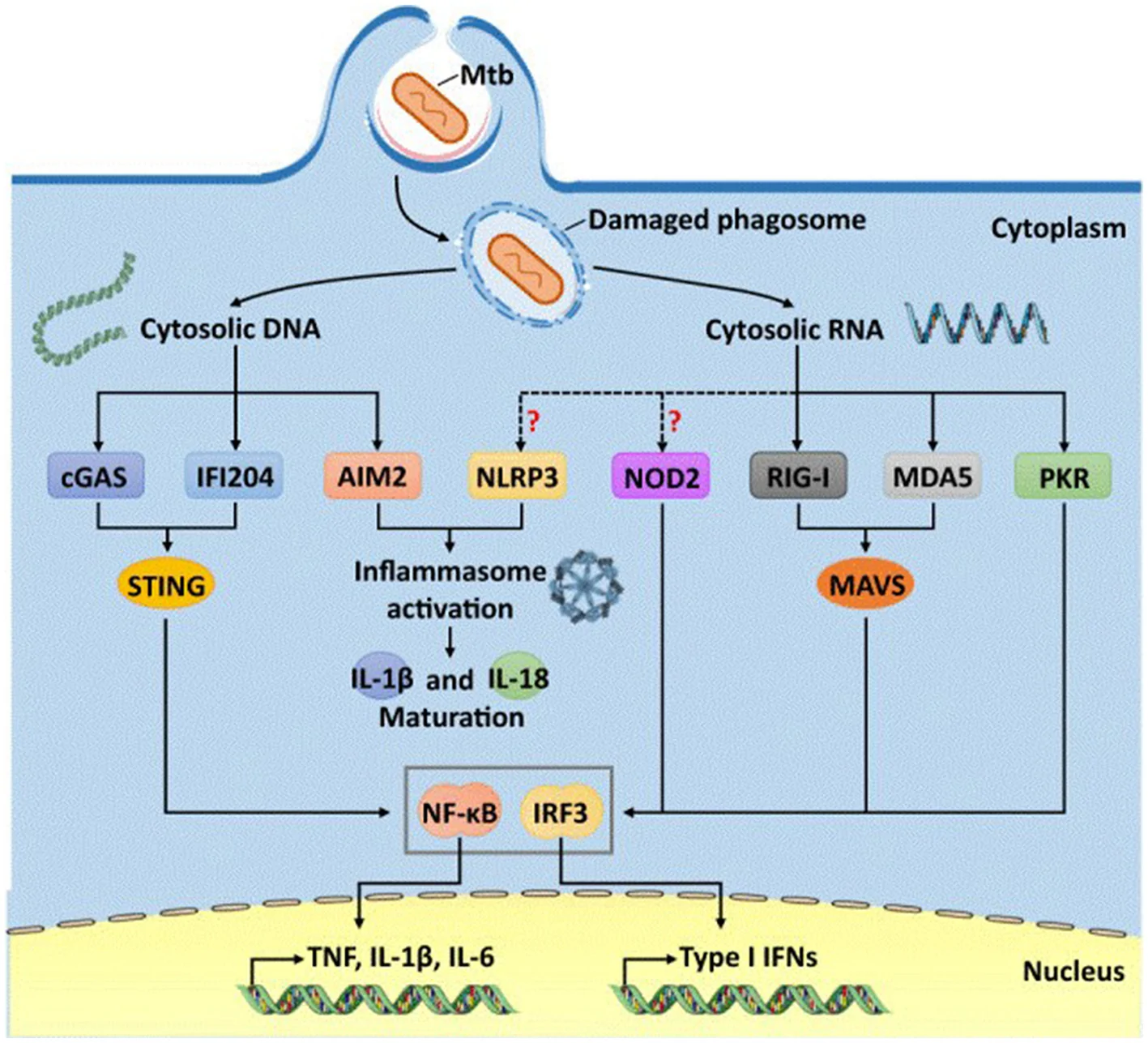
Cytosolic DNA- and Mtb RNA-sensitive pathways for immune recognition of *Mycobacteria tuberculosis* (13).

Currently, there is no clear understanding changes in the immune response is fundamental in developing new methods for preventing, diagnosing, and treating tuberculosis infection (Figure 1).

At the same time, questions remain regarding the mechanisms of transition of latent tuberculosis infection (LTBI) to active tuberculosis infection (17). Studying the dynamics of the transition from *M. tuberculosis* and pathogen multiplication to the development of inflammation in tissues is extremely difficult due to the characteristics of the pathogen and the lack of a clear understanding of the possibility of the duration of mycobacterial persistence in the human body (18).

Under experimental conditions, the dynamics of TB of lungs and lymph nodes were analyzed in the macaque model. When Cynomolgus macaques were infected by *M. tuberculosis*, it was shown that in 50% of cases macaques became ill with active tuberculosis, but in the other half of the animals latent tuberculosis infection was determined (19).

At the same time, Rhesus macaques are more susceptible to *M. tuberculosis*. They always develop active tuberculosis when infected with a virulent strain of *M. tuberculosis* (20).

It has been shown that if TB infection the lymph nodes, an adaptive immune response occurs. The primary mechanism of bacterial killing is probably related to the production of cytokines, chemokines, cytolytic and other effector molecules by lymph node cells (21).

The results of experimental studies on Cynomolgus macaques have brought us closer to understanding the development of tuberculosis infection and, most importantly—which is practically impossible to do in humans—latent tuberculosis infection and allowed us to assess the immune response to specific *M. tuberculosis* antigens and the efficacy of preventive chemotherapy in individuals with LTI. These mechanisms cause its ineffectiveness, in particular in widespread granulomas of thoracic LU, which will allow us to find ways to solve the problems of treatment of preclinical tuberculosis infection. At the same time, understanding of changes in the immune response is one of the fundamental in the development of new methods of prevention, diagnosis and treatment of tuberculosis infection.

## 3 *M. tuberculosis* and cells innate immunity

Numerous surface determinants or pathogen associated molecular patterns (PAMPs) of *M. tuberculosis* have now been described that are efficiently recognized by various pattern-recognizing receptors of the innate immune system. It should be noted that the spectrum of these pattern-recognizing receptors is broad and includes C-type lectins (e.g., mannose receptor, DC-SIGN, Dectin-1, Dectin-2, etc.), receptors for components of the complement cascade (e.g., CR3), and various collectins (sulfactant proteins A and D, mannose-binding lectin), scavenger receptors or skevenger receptors (including MARCO, SR-A1, CD36, SR-B1), as well as various types of receptors for immunoglobulin class D (Fcg receptors) and Toll-like receptors (including TLR-2, TLR-4, TLR-9). Among the PAMPs recognized by these receptors, which can be located both on the cell membrane surface and as part of the endosomes and cytosol of cells, PAMPs from the cell wall and cytoplasm of *M. Mannosylated lipoarabinomannan* (ManLAM), *phosphatidylinositolmannosides* (PIM), *phthioceroldimicocerosates* (PDIM), *phenolic glycolipids* (PGL), *trehalosodimicolate* (TDM), peptidoglycan, and several others are among the most recognized of these receptors (4).

During infection, *M. tuberculosis* is taken up by phagocytic cells of the host. *M. tuberculosis*-induce damage to phagosomes releases bacterial DNA and RNA into the host cytosol. The cytosolic sensors GAS, IFI204 and AIM2 recognize *M. tuberculosis* DNA, whereas RIG-I, MDA5 and PKR detect RNA. Although NLRP3 and NOD2 also respond to Mtb infection, it remains unclear whether they are directly activated by *M. tuberculosis* RNAs.

TLR2 forms heterodimers with either TLR1 or TLR6. These heterodimers are involved in the recognition of mycobacterial cell wall glycolipids such as LAM, a mycobacterial glycoprotein, and PIM, triacylated (TLR2/TLR1) or diacylated (TLR2/TLR6) lipoproteins. TLR2 is thought to play an important role in the induction of host innate immunity responses through activation of TNFα expression and secretion in macrophages. In turn, TLR2 and TLR6, but not TLR4 or TLR9, play an important role in stimulating IL-1β production. TLR4 is activated by heat shock proteins 60/65, which are secreted by different strains of *M. tuberculosis*. In turn, TLR9 recognizes unmethylated CpG repeats in bacterial DNA (5).

*M. tuberculosis* DNA and some secondary messengers (e.g., cyclo-diadenosine monophosphate) can be recognized by cytosolic PRRs such as cGAS and STING, resulting in the triggering of cytokine production and induction of autophagy in the infected cell. In addition, NOD-like receptors (from the English “nucleotide oligomerization domain-like receptors” or NLRs), localized in the cytosol of most cells in the body, recognize *M. tuberculosis* PAMPs such as muramyl dipeptide and cause activation of a multiprotein complex called the “inflammasome”. Inflammasome is sometimes considered as the main signaling pathway for activation of antimycobacterial host defense, since it is this complex, through caspase-1 activation, leads to the processing (or limited proteolysis) of proform cytokines of the IL-1 family—IL-1β and IL-18, which have pronounced proinflammatory activity.

In addition, *M. tuberculosis* actively secrete several compounds that induce the expression of genes encoding type I interferons (IFN-I), which include double-stranded DNA and the bacterial secondary messenger cyclic di-AMP (6). These compounds are recognized by various cytosolic PRRs including cGAS, IFI-204, AIM2 and possibly NOD2. Activation of these cytosolic PRRs leads to the activation of STING (from the Stimulator of Interferon Genes), which subsequently forms a complex with TANK-binding kinase-1. This STING-TBK1 complex activates the transcription factor IRF3, which leads to the production of IFN-β in mice, as well as activation of human dendritic cells with subsequent production of type I IFN.

Mice defective in IRF3 produce IFN-β poorly, but are more resistant to M. tuberculosis infection, indicating a negative role of IFN-I in the pathogenesis of tuberculosis. The main effects of IFN-I during the infectious process caused by *M. tuberculosis* include the following: IFN-I mediate the recruitment of myeloid cells to the focus of pathogen entry (primarily population of monocytes/macrophages with pro-inflammatory activity, as well as DCs); IFN-I suppress the production and effects of IL-1β by these cells, which is essential for the initial stages of the host immune response to the microbacterium; prolonged production of IL-1β in the focus of inflammation is accompanied by destruction of surrounding tissues by activated cells, whereas type I IFNs and IFNγ can be considered as factors blocking the uncontrolled development of the inflammatory response. In addition, type I IFNs are able to influence IL-12 production by activated DCs. Thus, the excess of these cytokines in the environment can suppress IL-12 production, which reduces the efficiency of type I response development and “polarization” of Th0 toward Th1. Moreover, against the background of high concentrations of type I IFN, activated DCs are unable to respond to IFNγ, but enhance the production of anti-inflammatory IL-10. Thus, it prevents further IL-12 production by dendritic cells, inhibits their activation by IFNγ and leads to the “*M. tuberculosis-permissive*” phenotype (6, 7).

Thus, the “functional redundancy” of these multiple receptors probably allows a wide range of these PRRs to efficiently recognize a wide range of ligands within *Mtb*, which is accompanied by activation of various cells in the focus of pathogen invasion. Activation of multiple innate immune receptor signaling pathways simultaneously leads to a number of subsequent cellular antimicrobial responses, including enhanced phagocytosis and apoptosis. However, these defense responses can be modulated by *Mtb*,, which may provide some of the pathogen's cells with long-term intracellular survival (5, 8).

**Figure 2:**
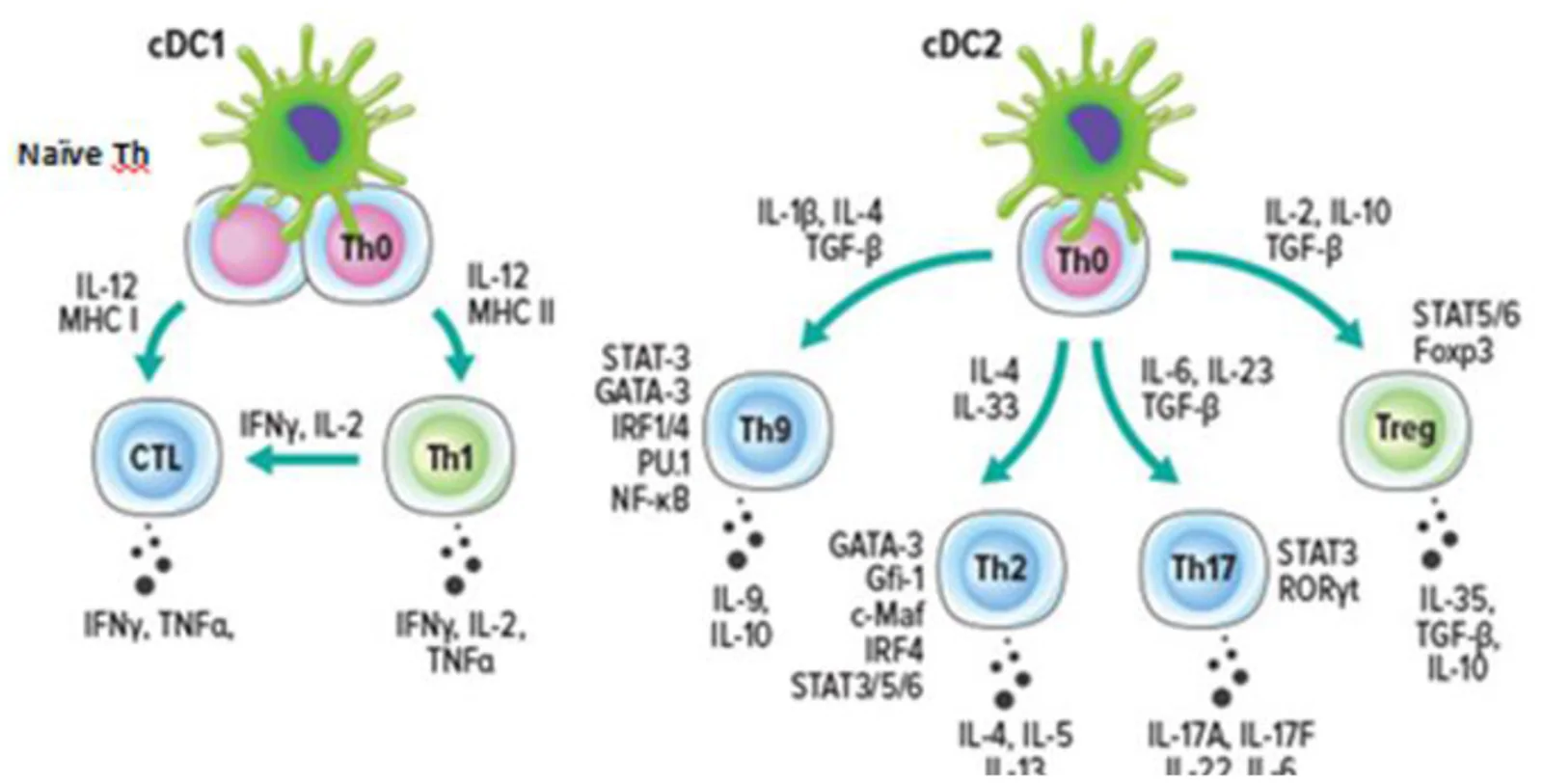
Dendritic cells and the “polarization” of T helper cells (3).

It should be recalled that dendritic cells circulating in the peripheral blood are heterogeneous population of leukocytes, which are traditionally divided into myeloid orCD123-CD11c+ (cDC, “conventional dendritic cell”) and CD123+CD11c-plasmacytoid dendritic cells (pDC, “plasmacytoid dendritic cell”) dendritic cells. cDC, in turn, are usually divided into two main subpopulation—cDC1 and cDC2, which differ both in their phenotype and functions. Thus, cDC1 cells carry BDCA-3 (CD141), Clec9A, CADM1, BTLA and CD26 on their membrane, and are also capable of cross-presentation of antigens by cytotoxic T-lymphocytes and “polarization” of “naive” T-helper cells toward Th1 (Figure 2) (3).

Whereas cDC2 have a CD1c+ phenotype (as well as FcεR1+SIRPA+) and play a leading role in the initiation of the response mediated by T-helper cells of various types. In recent years, two cell types have been distinguished within cDC2– CD5+ DC2 and CD5– DC3, with the marker CD14 also found on the surface of DC3 (22, 23).

Within type 1 inflammation, pDCs, with a CD123+CD11c– phenotype, play a pivotal role through the production of type I and III IFNs, as well as IL-12, necessary for Th1 “polarization” and ILC1 activation. Importantly, pDCs carry on their surface histocompatibility class II (MNS-II) and co-stimulatory molecules CD40, CD80 and CD86, which are necessary for antigen presentation to CD4+ T-lymphocytes (8). However, the antigen-presenting function of pDCs is not as efficient as that of DCs (cDCs). Expression of major histocompatibility complex class I and II molecules (MHC I and MHC II) together with co-stimulatory molecules (CD80, CD86 and CD40) allow pDCs to perform antigen presentation to both CD8+ T-lymphocytes and CD4+ T-helper cells. Whereas the secretion of type I IFN and IL-12 by pDCs is able to stimulate proliferation, differentiation and maturation of effector CD8+ T-lymphocytes, as well as differentiation and “polarization” of “naive” CD4+ T cells toward Th1. Moreover, the pDCs population is one of the main sources of type I interferons (α, β) at the early stages of immune response development (9). These cells due to the expression of TLR-7 and TLR-9 in endosomes are able to recognize viral nucleic acids—single-stranded RNA (ssRNA) and CpG sequences within DNA. The fact that pDCs activated through TLRs produce 100–1,000 times more type I IFN than ordinary somatic cells of the organism deserves special attention, which determines the most important role of these cells in the formation of signals necessary for the initiation of antiviral response.

When analyzing the peripheral blood of patients infected with *Mtb*, decreased levels of both cDC and pDC were shown compared to healthy donors. Another study showed that the amount of cDC (HLA-DR+CD11c+CD123–) was reduced in the blood of patients with pulmonary tuberculosis compared to patients with pleural tuberculosis, who have a better prognosis. A significant decrease in IL-12-producing mDCs was observed in patients with pulmonary TB compared with mDCs in patients with pleural TB and in healthy subjects. *In vitro* infection of human DCs with *M. tuberculosis* or BCG also induce production of the anti-inflammatory cytokine IL-10 and impaired IL-12 secretion and type I IFN production. In addition, it was shown that some proteins secreted by *M. tuberculosis* or included in mycobacterial coverslips were able to modulate the activity of dendritic cells, which allows us to consider them as one of the mechanisms of immune response avoidance (24–26).

For example, under *in vitro* conditions, Man-LAM significantly suppressed IL-12 secretion in human DCs pre-activated by LPS. Man-LAM also inducepartial maturation of DCs compare to LPS-induce maturation. Man-LAM also induces partial maturation of DCs compared to LPS-induce maturation. As mentioned above, Man-LAM is a ligand for the mannose receptor and DC-SIGN, which are presented on the surface of DCs. The interaction between Man-LAM and DC-SIGN promoted the uptake of live *M. tuberculosis* and prevented LPS-induce DC maturation. Notably, inhibition of this interaction using DC-SIGN-blocking monoclonal antibodies reduced *M. tuberculosis* uptake by 90%. In addition, the interaction between BCG and DC-SIGN induce uptake of live *M. tuberculosis*, partial DC maturation and IL-1 production. Notably, BCG cell wall components induce the maturation of human DCs through the secretion of TNFα and IL-12, which was dependent on the activation of TLR2 and TLR4. Moreover, recognition of *M. tuberculosis* through signaling from TLRs led to effective development of a proinflammatory response, whereas binding of bacteria to DC-SIGN or mannose receptor on the surface of human dendritic cells was accompanied by triggering IL-10 production and development of an anti-inflammatory response by DCs.

Thus, another way of avoiding the immune response used by *M. tuberculosis* is switching the synthesized cytokines by dendritic cells from IL-12, which promotes the development of type 1 inflammation, to IL-10, which has pronounced immunosuppressive properties. All this is accompanied by a decrease in the efficiency of development of specific immune response mediated by *M. tuberculosis*-specific Th1 cells. Moreover, in addition to blockade of IL-12 production, the ability of *M. tuberculosis to* suppress the expression, formation of active forms and secretion of IL-18, the second most important cytokine that ensures the formation of Th1 cells, was noted. Natural killer cells (NK cells) belong to the system of innate immunity, are able to secrete IFNγ and perform cytolytic functions through the induction of apoptosis in target cells, which allows them to control various intracellular pathogens, including *M. tuberculosis* (22). Various components of the *M. tuberculosis* cell wall can directly bind to the NKp44 receptor of NK cells, and NK cells can independently recognize cell stress molecules, the expression of which is increased on the surface of *M. tuberculosis* cells. NK cells, due to their cytolytic properties, can perform direct killing of *M. tuberculosis*-infected macrophages, but can also limit intracellular bacterial replication by secreting IL-22 and IFNγ, which increase the fusion efficiency of lysosomes and phagosomes containing *M. tuberculosis*. In addition, NK cells can enhance immunity against *M. tuberculosis* indirectly by increasing IFNγ production by CD8+ T cells, promoting proliferation of γδ T cells and killing regulatory T cells (23, 24).

## 4 Activation of the acquired immunity system in tuberculosis

In *M. tuberculosis* infection, the key effector T-helper cells are Th1 cells, which, upon recognition of a specific antigen in peripheral tissues, are able to produce IFNg, which activates the widest range of immunocompetent cells, including CD8+ cytotoxic T-lymphocytes, ILC1, macrophages, and B-cells that participate in the elimination of pathogens localized inside cells (25–27). It is now known that the infectious process caused by *M. tuberculosis* induces the differentiation of various subpopulations of T-helper cells other than Th1. It has also been shown that maturation of monocyte-derived DCs during *M. tuberculosis* infection induces IL-17 production by autologous CD4+ T cells. It is important to emphasize that activation of the dectin receptor involved in mycobacterial recognition increased the production of both IL-17 (the main effector cytokine Th17) and IFNg (the main effector cytokine Th1) (24, 28).

In contrast, activation of mannose receptor or DC-SIGN by Man-LAM *M. tuberculosis* significantly reduced IL-17 production but stimulated IFNg release by cells, although the exact mechanisms of this process are still poorly understood. *In vitro* experiments, it was shown that the presence of *M. tuberculosis* during the differentiation of dendritic cells of monocytic origin promoted a more efficient “polarization” of Th0 toward Th2 and Th17 when compared to mature DCs stimulated with *M. tuberculosis*. Moreover, the preferential formation of Th2 and Th17 from Th0 was associated with reduced expression of DC-SIGN and mannose receptor by dendritic cells against a background of high expression of the Dectin-1 receptor. Thus, differentiation of Th2 and Th17 to the detriment of Th1 may represent another immune response evasion strategy employed by *M. tuberculosis*. It should also be noted, ESAT-6 protein under *in vitro* conditions suppressed DC maturation induceby LPS and CD40L stimulation, decreased T cell proliferation and IFNg production, but increased IL-17 production. There is also evidence that such *M. tuberculosis* antigen as Rv1917c enhanced dendritic cell production of IL-10 under *in vitro* conditions and promoted “polarization” of Th0 toward Th2 (16).

In 2019, it was shown that in TB patients in peripheral blood the relative content of Th2 cells significantly increased and the level of Th17 significantly decreased, while no significant differences between groups were observed in the case of Th1 and Tfh cells (20). Similar results were obtained when analyzing the subpopulation composition of Th cells in peripheral blood in TB using nonspecific stimulation methods *in vitro*, when it was shown that the level of CD4+IL-17A+ cells decreased against the background of infection, while the content of CD4+IL-4+ lymphocytes in patients significantly increased (24). However, there are also diametrically opposite observations, when Wang et al. found an increased content of IL-17+CD4+ cells in circulation in TB patients compared to the control group, which confirmed previously published studies on the increased level of IL-17 mRNA in peripheral blood lymphocytes of patients with active TB (29, 30).

On the other hand, two independent groups of researchers noted a decrease in Th17 levels in the peripheral blood of patients. Moreover, decreased IL-17 levels in the peripheral blood of TB patients were closely associated with low efficacy of the applied therapy and unfavorable outcome of the disease. Disruption of T-helper polarization is accompanied by a change in the pattern of effector cytokines that are secreted in the foci of pathogen penetration, which has a critical impact on the effector phase of the immune response. For example, the Th1 cytokine IFNg can induce macrophage autophagy, which promotes more efficient elimination of mycobacteria, whereas the Th2 cytokines IL-4 and IL-13 abolish the enhancement of autophagy, which interferes with the destruction of intracellular mycobacteria and promotes their survival within infected cells.

### 4.1 Th1 cells

During the development of the adaptive immune response, after TCR activation in a specific cytokine microenvironment, “naive” CD4+ T cells can differentiate or “polarize” into Th1 cells. A crucial role in the polarization of Th0 into Th1 cells is played by IL-12, which can be secreted by APCs in response to recognition of certain PAMPs. IL-12 activates the transcription factor STAT4. At the same time, IFNγ produced by NK cells activates another transcription factor, STAT1. Simultaneous activation of expression of STAT1 and STAT4 transcription factors in Th0 lymphocytes can induce expression of the main Th1-polarizing factor T-bet. T-bet, together with other transcription factors (Hlx, Runx3, Ets-1 and Bhlhe40), triggers the expression of the gene encoding IFNγ (31, 32).

T-bet, alone or in combination with Runx3, inhibits the expression of the transcription factors GATA3 and RORγt, which control the polarization of Th0 into other types of Th (Th2 and Th17). Recently, IL-23 and IL-27 have also been found to have the ability to “polarize” Th0 into Th1. Not only “naive” CD4+ T lymphocytes are capable of polarization into Th1. Due to the significant “plasticity” of polarized helper cells, Th1 can originate from other T helper subpopulations, including Th17, Treg, and Tfh cells. Th1 express a characteristic set of chemokine receptors that regulate their migration into the inflammatory focus. The main chemokine receptor of Th1 cells are CXCR3+ and CCR5, which determines the ability of these cells to migrate along the concentration gradient of chemokines CXCL9, CXCL10 and CXCL11– ligands of CXCR3+ and the concentration gradient of CCL3, CCL4 and CCL5– ligands of CCR5. Under the influence of IFNγ, keratinocytes and epithelial cells produce CXCL9, CXCL10 and CXCL11, which contributes to additional recruitment of Th1 cells to the focus of pathogen invasion and enhancement of type 1 inflammation. High levels of these three chemokines have been reported in the circulating blood of patients with tuberculosis (31), with serum CXCL9 levels correlated with systemic organ involvement and CXCL10 levels strongly correlated with respiratory outcomes in patients infected with Mtb (32). In *Tbc*, high levels of CXCL9 and CXCL10 have been reported in patients with active pulmonary TB and latent TB infection (33, 34).

The chemokine receptor CXCR3+ is thought to play an important role in T cell migration into lung during TB (35). Moreover, a mouse model of TB showed ~three-folds more number of CD4+ CXCR3+ T cells and ~six-folds more CD8+ CXCR3+ T cells in lung airway lumen compared to controls (36). Whereas, in the peripheral blood of patients with active pulmonary tuberculosis, there was an increase in the subpopulation of CD4+ CXCR3+ cells, which can be considered as Th1 cells (37). It was also recently shown that the level of CXCR3+ cells in TB patients increased in all major subpopulation of Th cells at different stages of maturation, including CD45RA-CD62L+ central and CD45RA-CD62L– effector memory cells, as well as mature effector cells of the TEMRA population (CD45RA+CD62L–).

Moreover, the increased content of CXCR3+ TEM and CXCR3+ TCM compared to the peripheral blood of the same patients was noteworthy in the BAL of patients with pulmonary tuberculosis (38). Probably, CXCR3+ *M. tuberculosis*-specific Th1 cells are able to migrate to the foci of infection. Nikitina et al. observed that lung CD4+ T-lymphocytes in active pulmonary tuberculosis resembled CXCR3+CCR6– population and predominate in lung tissue (39). Thus, Th1 in tuberculosis contribute to granuloma formation and limit the spread of TB.

### 4.2 CD8+ T cells

The main function of CD8+ T-lymphocytes is to destroy infected or tumor target cells. These cells realize their effector potential by three main mechanisms. The first mechanism is based on the production of effector cytokines IFNγ and TNF upon recognition of target cells via the T cell receptor. The second mechanism is “contact cytolysis” based on the release of perforin and granzymes from granules, as previously described for NK cells. Finally, the third way to kill target cells is to trigger apoptosis by activating receptors belonging to the TNF-like protein family on their surface (the classic example is the Fas/FasL interaction).

Similar to CD4+ Th1 differentiation, IFNγ and IL-12 promote the differentiation and maturation of cytotoxic CD8+ T lymphocytes. The transcription factor T-bet activates in cytotoxic T cells to produce IFNγ and is also essential for their cytolytic potential. Moreover, CD8+ T-lymphocytes (primarily Tc1) express on their surface the chemokine receptors CCR5 and CXCR3+, which are also characteristic of Th1, allowing these two T cell population to migrate in a directed manner to the same inflammatory focus localized in peripheral tissues. The importance of CD8+ T cells in *M. tuberculosis* infection is related to their ability to secrete cytokines and cytolytic effector molecules that can limit bacterial replication. In addition to IFNγ and TNFα, CD8+ T cells secrete perforin to induce apoptosis in *M. tuberculosis*-infected macrophages. In addition, CD8+ T cells can also release granulysis in from cytotoxic granules to directly kill intracellular *M. tuberculosis*.

Recently, we have shown that Tc1 (CCR6-CXCR3+) CD8+ T-cells decreased in the peripheral blood of patients with tuberculosis, which may be associated with the directed migration of these cells to the foci of inflammation, where ligands for the chemokine receptor CXCR3+ are actively expressed (40). Similar results were obtained by Alduenda et al. (41) who found a decrease of CXCR3+CD8+ Tcells in the circulation in patients without therapy, whereas administration of therapy was accompanied by a restoration of the pool of these cells in the peripheral blood. This is also supported by the fact that CD8+ Tcells are detected in the walls of alveoli and pulmonary cavities on immunohistochemical examination of biopsy specimens in pulmonary tuberculosis (42), which may indicate effective migration of CXCR3+CD8+ T cells into the focus of inflammation. In experiments on Cxcr3(–/–) mice, the crucial role of CXCR3+ CD8+ T cells in defense against intracellular pathogens was also demonstrated (43). In addition, in patients with tuberculosis-associated immune reconstitution inflammatory syndrome (TB-IRIS) infected with both TB and HIV, a decrease in the proportion of CXCR3+ CD8+ T-cells was observed (44). Moreover, an inverse correlation between the content of “naïve” CXCR3+ CD8+ T cells and the risk of TB-IRIS development was found, whereas an increase in the level of CXCR3+ effector CD8+ T cells was closely associated with the probability of TB-IRIS development.

Th17 cells play a leading role in the development of the type 3 antigen-specific response aimed at defense against extracellular bacteria and fungi and are involved in the development of many autoimmune diseases, including multiple sclerosis (10, 11). Several independent studies analyzing the role of IL-23 and IL-12 in the development of autoimmune pathologies, published in 2003, served as a basis for the designation of Th17 cells as a separate type as early as 2005. Bacterial and fungal cell wall components can activate APCs to produce proinflammatory cytokines IL-1β, IL-6, and IL-23, which in turn direct “naive” CD4+ T cells to differentiate toward Th17.

Interestingly, more regulatory molecules are required for Th0 differentiation and “polarization” into Th17 than for “polarization” into other helper types. The cytokines IL-6 and IL-23 activate STAT3, which is required to trigger expression of the major Th17-polarizing transcription factor RORγt. TCR signaling can also induce RORγt expression indirectly, through activation of NFAT/NF-κB/AP-1. In turn, RORγt, along with other transcription factors (IRF4, BATF and Runx1), promotes the activation of Th17 cell-specific gene expression. TGF-β in combination with IL-6 and IL-21 are involved in the differentiation of Th17 cells under *in vitro* conditions, although the possibility of a TGF-β-independent pathway of Th17 cell differentiation has been reported in the literature. Th17 secrete effector cytokines, IL-17A, IL-17F, and IL-22, which induce the secretion of matrix metallopeptidases (MMP), nitric oxide (NO), cytokines and antimicrobial peptides in different types of immune and non-immune cells. Th17 cells also play an important role in defense against *M. tuberculosis* (45).

In analyzing the subpopulation composition of Th peripheral blood in TB with the use of nonspecific stimulation methods *in vitro* conditions. It was shown that the level of CD4+IL-17A+ cells in the circulation decreased against the background of infection, while the content of CD4+IL-4+ lymphocytes in patients significantly increased (46). Thus, Wang et al. found an increased IL-17+CD4+ cell content in the circulation in TB patients compared to controls (47), which confirmed the previously published data on the increased IL-17 mRNA level in peripheral blood lymphocytes of patients with active TB (48). On the other hand, two independent groups of researchers noted a decrease in the level of Th17 in the peripheral blood of patients (48, 49).

It was noted that the decrease in circulating Th17.1 cells and CCR6+DP Th17 cells in the peripheral blood of patients significantly increased compared to the control group (45, 50). On the other hand, the accumulation of IFN-γ+IL-17+ Th17.1 cells correlated with the disease severity, and the maximum levels of these cells were observed in low responder TB patients, individuals displaying severe pulmonary lesions, and longest length of disease evolution (51). It is also worth mentioning the work of Marín et al. (52) who showed that in patients with active TB during *in vitro* stimulation with PPD the level of IL-17-producing CD4+ T cells exceeded the values of patients with LTB, while in patients with LTB the level of IFN-γ-producing CD4+ T cells was higher than in healthy controls.

### 4.3 Follicular T cells (Tfh)

Follicular T cells (Tfh) play a crucial role in the maturation and differentiation of B cells within the germinal center reaction in peripheral lymphoid organs (53). These cells control the processes of switching the classes of antibodies synthesized by the B cells, triggering somatic hypermutations, and selecting high-affinity B cells clones that further differentiate into plasma cells and memory cells. Currently, follicular T-helper cells (Tfh) circulating in the peripheral blood are considered as a heterogeneous cell population, within which, based on the co-expression of chemokine receptors CCR6 and CXCR3+, as well as the presence of various transcription factors peculiar to “polarized” T-helper subpopulation, they can be divided into several independent groups (54). Thus, Th1-like Tfh or Tfh1 carry only CXCR3+ on their surface, and the transcription factor Tbet is found in their nuclear compartment. Whereas Th2-like Tfh or Tfh2 are negative for both of the above chemokine receptors but express high levels of the transcription factor GATA3. Finally, Th17-like Tfh or Tfh17 have a CXCR3+ CCR6+RORγt+ phenotype (50, 54).

It is believed that the balance between different Tfh population is the basis for effective functioning of the humoral link of acquired immunity, whereas its shift—primarily, an increase in the level of cells capable of stimulating B cells (population with Tfh2 and Tfh17 properties)—may be accompanied by the development of autoimmune reactions and chronic inflammation. Thus, using experimental models on laboratory animals, it was shown that IFNg-secreting Th17 negatively affect the formation of long-term immune defense against repeated *M. tuberculosis* infiltrations, whereas accumulation of CXCR5+ Th17 in lung tissue increases the efficiency of protective reactions at least in mice (55). Immunohistological analysis of granulomas in experimental infection of laboratory animals (56), as well as samples obtained from primates (57) and patients infected with *M. tuberculosis* (58) revealed the presence of decorated clusters of lymphocytes, which included not only CD3+ but also CD19+ lymphocytes, forming structures resembling germinal centers of lymph nodes and containing proliferating B-cells. Moreover, the formation of highly organized ectopic lymphoid clusters containing CXCR5+ T cells in granulomas was closely associated with a favorable outcome in LTBI, whereas poorly organized or diffuse lymphoid masses with low levels of Tfh cells were formed in active TB and had poorly organized Tfh cells (25).

The latter study also showed that CD4+CXCR5+ lymphocytes were capable of IFNg production, which allowed us to consider them as Tfh1. During *Tbc*, we observed a change in the subpopulation composition of Tfh associated with an increase in the circulating Tfh2 with CXCR3-CCR6- phenotype against the background of a decrease in the relative content of CXCR3+CCR6– Tfh1 cells, while no significant changes in the percentage of the remaining two types of Tfh– Tfh17 and “double-positive” Tfh– were observed (50, 59). Disturbances in the functional activity of circulating Tfh cells were observed in a study conducted by Kumar et al. on the peripheral blood of patients with active TB (59). It was shown that not only the level of IL-21-producing cells in the blood of patients was reduced relative to control values, but also the concentrations of this cytokine in the systemic bloodstream in the case of infection with mycobacteria were lower than those of the comparison group. Moreover, the level of IFNg, the production of which is traditionally associated with the Tfh1 cell population, was also reduced in patients, which confirms the results we obtained on changes in the subpopulation of Tfh lymphocytes in TB patients.

## 5 Risk factors and mechanism of LTBI reactivation

The mechanism of reactivation of the tuberculosis process is caused mainly by damage to the mechanisms of immune containment of *M. tuberculosis*. Some of the key cytokines that inhibit the dissemination of *M. tuberculosis* are TNFα and IFNγ. Both of these cytokines are involved in type 1 T cell immune response. TNFα promotes activation of macrophages (59) in the lesion site, granuloma formation (60), initiation of apoptosis of infected cells (61), as well as the migration of predominantly CXCR3+T cells to the site of infection through the induction of CXCL9 (MIG) and CXCL10 (IP-10) (62). IFNγ has similar functions. It also promotes macrophage activation and tuberculous granuloma formation (63). In addition, IFNγ can prevent *M. tuberculosis* from blocking phagolysosome assembly by inducing apoptosis of the infected cell (64). Disruption of production of these cytokines, as well as defects in their signaling cascades, can contribute to reactivation of tuberculosis infection. Thus, Scanga et al. in their study demonstrated the transition of latent tuberculosis infection to active upon blocking TNFα in a modified Cornell murine model (65). There are also numerous reports on the development of tuberculosis in patients using anti-TNFα therapy (66, 67), which requires preliminary diagnosis of LTBI before starting to use these drugs. *In vitro* TNFα blockade models have demonstrated negative modulation of Th1 and Th17, as well as marked changes in tuberculous granuloma structure (68). Similar to TNFα blockade, IFNγ neutralization can also lead to reactivation of latent tuberculosis infection, as shown in mouse models (65, 69). Moreover, recently there have been reports on the positive effect of adding IFNγ to the therapy of patients with tuberculosis. Thus, Gao et al. in their meta-analysis, including nine clinical trials, demonstrated a statistically significant effect of IFNγ as an adjunctive drug for the treatment of tuberculosis infection (70).

The main cells producing TNFα and IFNγ are Th1 and Tc1, and the main effector cells influenced by the above-mentioned cytokines are macrophages. Foreman et al. demonstrated in their study that increased proliferation of granzyme B-expressing memory CD8+ T cells correlates with protection against reactivation of tuberculosis infection (71). Accordingly, drugs and diseases that cause a decrease in these cells can cause LTBI reactivation. For example, in HIV infection, there is a decrease in CD4+ T cells in the peripheral blood, which contributes to an increased risk of reactivation of tuberculosis infection (72). In the case of diabetes mellitus, there is a violation of monocyte migration and adhesion, as well as a predominance of the anti-inflammatory phenotype of macrophages (73). Thus, diabetes mellitus increases the risk of active tuberculosis (74). The use of an immunosuppressant such as azathioprine requires alertness regarding the development of active tuberculosis disease. Thus, it has been proven that in patients with IBD, the risk of developing tuberculosis increases when azathioprine is prescribed (75). In addition, when using this drug in patients with dermatomyositis, the risk of tuberculosis infection also increases (76). Also, the use of immunosuppressants in kidney, liver, and heart transplantation increases the risk of tuberculosis (77). Chronic kidney disease (CKD) may contribute to immunosuppression (78). It has been shown that patients with CKD have decreased proliferation (79) and increased apoptosis of CD4+ and CD8+ T cells (80). Thus, patients with CKD have an increased risk of *M. tuberculosis* infection due to immunosuppression, which has been proven by numerous studies (81–83).

The cytokines TNFα and IFNγ, as well as Th1, Tc1 and macrophages are mainly involved in protection against reactivation of tuberculosis infection. In various immunodeficiency states, the pool of the above-mentioned cells is depleted, the effective synthesis of TNFα and IFNγ decreases, which leads to reactivation of tuberculosis. Thus, various immunodeficiency states, such as HIV infection, diabetes mellitus, CKD, immunosuppressants are risk factors for LTBI reactivation and require special vigilance from clinicians. In turn, the insufficiency of TNFα- and IFNγ-positive T cells in circulation can act as a predictor of the transition of latent tuberculosis infection to active disease.

According to the studies conducted, TB lymph nodes, which activates the adaptive immune response. The primary mechanism of bacterial killing is probably related to the production of cytokines, chemokines, cytolytic and other effector molecules by lymph node cells (21). Activation of multiple innate immunity receptor signaling pathways simultaneously leads to a number of subsequent cellular antimicrobial responses, including increased phagocytosis and apoptosis. However, these defenses responses can be modulated by *M. tuberculosis*, which may ensure long-term intracellular survival of a part of the pathogen cells. There are ways to avoid triggering an immune response. One of the ways used by *M. tuberculosis* is to switch the cytokines synthesized by dendritic cells from IL-12, which promotes type 1 inflammation, to IL-10, which has pronounced immunosuppressive properties. All this is accompanied by a decrease in the efficiency of development of specific immune response mediated by *M. tuberculosis*-specific Th1 cells. Moreover, in addition to blockade of IL-12 production, the ability of *M. tuberculosis* to suppress the expression, formation of active forms and secretion of IL-18, the second most important cytokine ensuring the formation of Th1 cells, was noted.

When the organism is infected with *M. tuberculosis*, the key effector T-helper cells are Th1 cells, which upon recognition of a specific antigen in peripheral tissues are able to produce IFNg, which activates the widest range of immunocompetent cells, including CD8+ cytotoxic T-lymphocytes, ILC1, macrophages and B-cells, participating in the elimination of pathogens localized inside the cells (27, 87). It is now known that the infectious process caused by *M. tuberculosis* induces differentiation of various T-helper subpopulation other than Th1. Maturation of dendritic cells during Tbc induces IL-17 production by autologous CD4+ T cells. It is important to emphasize that activation of the dectin receptor involved in mycobacterial recognition increased the production of both IL-17 (a major effector cytokine of Th17) and IFNg (a major effector cytokine of Th1). In contrast, activation of the mannose receptor or DC-SIGN by Man-LAM *M. tuberculosis* significantly decreased IL-17 production but stimulated IFNg release by cells, although the exact mechanisms of this process are still poorly understood (88). According to the studies conducted, Th17 cells play a key role in the development of type 3 antigen-specific response. Several independent studies analyzing the role of IL-23 and IL-12 in the development of autoimmune pathologies, published in 2003, served as a basis for distinguishing Th17 cells as a separate type already in 2005. Proinflammatory cytokines IL-1β, IL-6 and IL-23 produced under the influence of mycobacteria direct ‘naive' CD4+ T cells to differentiate toward Th17. The cytokines IL-6 and IL-23 activate STAT3, which is required to trigger expression of the major transcription factor Th17-polarizing RORγt. In addition, RORγt along with other transcription factors (IRF4, BATF and Runx1) promotes the activation of Th17 cell-specific gene expression. TGF-β in combination with IL-6 and IL-21 are involved in the differentiation of Th17 cells under *in vitro* conditions, although the possibility of a TGF-β-independent pathway of Th17 cell differentiation has been reported in the literature. Th17 secrete effector cytokines—IL-17A, IL-17F and IL-22, which induce the secretion of matrix metallopeptidases (MMP), nitric oxide (NO), cytokines and antimicrobial peptides in different types of immune and non-immune cells.

## 6 Conclusions

The COVID-19 pandemic has drawn even more attention to the problem of tuberculosis, it is associated with an increase in new cases of tuberculosis (6, 84). The development and introduction of new immunological methods of diagnostics of latent tuberculosis infection do not allow to answer the question about the transition of the organism into the state of active tuberculosis infection. A new concept of type 1, type 2 and type 3 immune reactions is presented, as well as tissue-driven reactions that play a crucial role in immune disorders or autoimmunity. Type 1 (Th1) immune reactions target intracellular pathogens such as *Mycobacterium tuberculosis* or viruses. CD4+ T cells (Th1), innate lymphoid cell type 1 (ILC1), natural killer (NK) cells, natural killer T cells (NK-T) and cytotoxic CD8+ type 1 (Tc1) lymphocytes detect and destroy infected cells and their contents. Interferon-gamma (IFN-γ) is the major effector cytokine and IgG1, IgG2, and IgG3 are the major antibodies. To date, there is no clear understanding of the mechanisms of latent tuberculosis infection after the organism encounters *M. tuberculosis* and further development of active tuberculosis infection (85, 86).

The action of all these factors is directed toward the elimination of extracellular pathogens. CXCL8 and G-CSF produced by Th17 attract neutrophils to sites of inflammation, which destroy pathogens by phagocytosis and production of a wide range of microbicidal substances. Human Th17 also synthesize IL-26, which can also be produced by Th1 cells. Perhaps future studies on the role of Th17 and cytokine levels, especially IL-6 and IL-21, may be useful in understanding the transition of LTBI to active TB.

## References

[1] Global Tuberculosis Report 2020. Geneva: World Health Organization (2020). p. 232. ISBN 978-92-4-001313-1.

[2] Tuberculosis surveillance and monitoring in Europe 2013 – 2019. Available at: www.ecdc.europa.eu (accessed October 15, 2020).

[3] WHO global lists of high burden countries for TB, multidrug/rifampicin-resistant TB (MDR/RR-TB) and TB/HIV, 2021–2025. (2021). p. 16. ISBN 978-92-4-002943-9 (accessed June 17, 2021).

[4] Migliori GB Thong PM Akkerman O Alffenaar JW Álvarez-Navascués F Assao-Neino MM. Worldwide effects of coronavirus disease pandemic on tuberculosis services January–April 2020. Emerging Inf Dis. (2020) 11:2709–12. 10.3201/eid2611.203163 32917293 PMC7588533

[5] McQuaid CF. The potential impact of COVID-19-related disruption on tuberculosis burden. Eur Respir J. (2020) 56:2001718. 10.1183/13993003.01718-2020 32513784 PMC7278504

[6] WHO consolidated guidelines on tuberculosis. Module 5: Management of Tuberculosis in Children and Adolescents. Geneva: World Health Organization (2022). 35404556

[7] Goudiaby MS Gning LD Diagne ML Dia BM Rwezaura H Tchuenche JM. Optimal control analysis of a COVID-19 and tuberculosis co-dynamics model. Inform Med Unlocked. (2022) 28:100849. 10.1016/j.imu.2022.100849 35071729 PMC8759807

[8] Glaziou P. Predicted impact of the COVID-19 pandemic on global tuberculosis deaths in 2020. medRxiv and *bioRxiv*. Consolidated guidelines on drug-resistant tuberculosis treatment. (2019), p. 96. ISBN 978-92-4-155052-9.

[9] Ntoumi F Nachega JB Aklillu E Chakaya J Felker I Amanullah F. World Tuberculosis Day 2022: aligning COVID-19 and tuberculosis innovations to save lives and to end tuberculosis. Lancet Infect Dis. (2022) 22:442–44. 10.1016/S1473-3099(22)00142-6 35248166 PMC8893724

[10] Annunziato F Romagnani C Romagnani S. The 3 major types of innate and adaptive cell-mediated effector immunity. J Allergy Clin Immunol. (2015) 135:626–35. 10.1016/j.jaci.2014.11.001 25528359

[11] Zhu X Zhu J. CD4 T helper cell subsets and related human immunological disorders. Int J Mol Sci. (2020) 21:8011. 10.3390/ijms21218011 33126494 PMC7663252

[12] Slogotskaya LV Litvinov V Ovsyankina E Seltsovsky P Kudlay D. Results of QuantiFERON-TB Gold in-tube and skin testing with recombinant proteins CFP-10-ESAT-6 in children and adolescents with TB or latent TB infection. Paediatr Respir Rev. (2013) 14:S565. 10.1016/S1526-0542(13)70092-7

[13] Kozlov VA Tikhonova EP Savchenko AA Kudryavtsev IV Andronova NV Anisimova EN. Clinical immunology. Practical guide for infectious disease specialists, Krasnoyarsk: Polikor. (2021). p. 550.

[14] Chai Q Wang L Liu CH Ge B. New insights into the evasion of host innate immunity by *Mycobacterium tuberculosis*. Cell Mol Immunol. (2020) 17:901–13. 10.1038/s41423-020-0502-z 32728204 PMC7608469

[15] Chai Q Lu Z Liu CH. Host defense mechanisms against *Mycobacterium tuberculosis*. Cell Mol Life Sci. (2020) 77:1859–78. 10.1007/s00018-019-03353-5 31720742 PMC11104961

[16] Hmama Z Peña-Díaz S Joseph S Av-Gay Y. Immunoevasion and immunosuppression of the macrophage by *Mycobacterium tuberculosis*. Immunol Rev. (2015) 264:220–32. 10.1111/imr.12268 25703562

[17] Lyadova I Nikitina I. Cell differentiation degree as a factor determining the role for different T-helper populations in tuberculosis protection. Front Immunol. (2019) 10:972. 10.3389/fimmu.2019.00972 31134070 PMC6517507

[18] Travar M Petkovic M Verhaz A. Type I, II, and III interferons: regulating immunity to *Mycobacterium tuberculosis* infection. Arch Immunol Ther Exp. (2016) 64:19–31. 10.1007/s00005-015-0365-7 26362801

[19] Plowes-Hernández O Prado-Calleros H Arroyo-Escalante S Zavaleta-Villa B Flores-Osorio J Ibarra Arce A. Cervical lymph node tuberculosis and TNF, IL8, IL10, IL12B and IFNΓ polymorphisms. New Microbiol. (2021) 44:24–32. 33582825

[20] Lyadova IV Panteleev AV. Th1 and Th17 cells in tuberculosis: protection, pathology, and biomarkers. Mediators Inflamm. (2015) 2015:854507. 10.1155/2015/854507 26640327 PMC4657112

[21] Wang X Zhang J Liang J Zhang Y Teng X Yuan X. Protection against *Mycobacterium tuberculosis* infection offered by a new multistage subunit vaccine correlates with increased number of IFN-γ+ IL-2+ CD4+ and IFN-γ+ CD8+ T cells. PLoS ONE. (2015) 10:e0122560. 10.1371/journal.pone.0122560 25822536 PMC4378938

[22] Abebe F. Immunological basis of early clearance of *Mycobacterium tuberculosis* infection: the role of natural killer cells. Clin Exp Immunol. (2021) 204:32–40. 10.1111/cei.13565 33315236 PMC7944356

[23] Sia JK Rengarajan J. Immunology of *Mycobacterium tuberculosis* infections. Microbiol Spectr. (2019) 7. 10.1128/microbiolspec.GPP3-0022-2018 31298204 PMC6636855

[24] Freches D Korf H Denis O Havaux X Huygen K Romano M. Mice genetically inactivated in interleukin-17A receptor are defective in long-term control of *Mycobacterium tuberculosis* infection. Immunology. (2013) 140:220–31. 10.1111/imm.12130 23721367 PMC3784168

[25] Slight SR Rangel-Moreno J Gopal R Lin Y Fallert Junecko BA Mehra S. CXCR5? T helper cells mediate protective immunity against tuberculosis. J Clin Invest. (2013) 123:712–26. 10.1172/JCI65728 23281399 PMC3561804

[26] Hilda JN Das S Tripathy SP Hanna LE. Role of neutrophils in tuberculosis: a bird's eye view. Innate Immun. (2020) 26:240–7. 10.1177/1753425919881176 31735099 PMC7251797

[27] Corleis B Dorhoi A. Early dynamics of innate immunity during pulmonary tuberculosis. Immunol Lett. (2020) 221:56–60. 10.1016/j.imlet.2020.02.010 32092359

[28] Rijnink WF Ottenhoff THM Joosten SA. B-cells and antibodies as contributors to effector immune responses in tuberculosis. Front Immunol. (2021) 12:640168. 10.3389/fimmu.2021.640168 33679802 PMC7930078

[29] Abreu R Giri P Quinn F. Host-pathogen interaction as a novel target for host-directed therapies in tuberculosis. Front Immunol. (2020) 11:1553. 10.3389/fimmu.2020.01553 32849525 PMC7396704

[30] Chung W Lee K Jung Y Kim Y Park J Sheen S. Serum CXCR3 ligands as biomarkers for the diagnosis and treatment monitoring of tuberculosis. Int J Tuberc Lung Dis. (2015) 19:1476–84. 10.5588/ijtld.15.0325 26614189

[31] Hasan Z Jamil B Khan J Ali R Khan MA Nasir N. Relationship between circulating levels of IFN-gamma, IL-10, CXCL9 and CCL2 in pulmonary and extrapulmonary tuberculosis is dependent on disease severity. Scand J Immunol. (2009) 69:259–67. 10.1111/j.1365-3083.2008.02217.x 19281538

[32] Arger NK Ho ME Allen IE Benn BS Woodruff PG Koth LL. CXCL9 and CXCL10 are differentially associated with systemic organ involvement and pulmonary disease severity in sarcoidosis. Respir Med. (2020) 161:105822. 10.1016/j.rmed.2019.105822 31783271 PMC7028429

[33] Kim S Lee H Kim H Kim Y Cho JE Jin H. Diagnostic performance of a cytokine and IFN-γ-induced chemokine mRNA assay after *Mycobacterium tuberculosis*-specific antigen stimulation in whole blood from infected individuals. J Mol Diagn. (2015) 17:90–9. 10.1016/j.jmoldx.2014.08.005 25528189

[34] Almeida CS Abramo C Alves CC Mazzoccoli L Ferreira AP Teixeira HC. Anti-mycobacterial treatment reduces high plasma levels of CXC-chemokines detected in active tuberculosis by cytometric bead array. Mem Inst Oswaldo Cruz. (2009) 104:1039–41. 10.1590/S0074-02762009000700018 20027475

[35] Jeyanathan M Afkhami S Khera A Mandur T Damjanovic D Yao Y. CXCR3 signaling is required for restricted homing of parenteral tuberculosis vaccine-induced T cells to both the lung parenchyma and airway. J Immunol. (2017) 199:2555–69. 10.4049/jimmunol.1700382 28827285

[36] Gupta A Saqib M Singh B Pal L Nishikanta A Bhaskar S. *Mycobacterium indicus pranii* induced memory T-cells in lung airways are sentinels for improved protection against Mtb infection. Front Immunol. (2019) 10:2359. 10.3389/fimmu.2019.02359 31681272 PMC6813244

[37] Orlando V La Manna MP Goletti D Palmieri F Lo Presti E Joosten SA. Human CD4 T-cells with a naive phenotype produce multiple cytokines during *Mycobacterium tuberculosis* infection and correlate with active disease. Front Immunol. (2018) 9:1119. 10.3389/fimmu.2018.01119 29875774 PMC5974168

[38] Li J Jing Q Hu Z Wang X Hu Y Zhang J. *Mycobacterium tuberculosis*-specific memory T cells in bronchoalveolar lavage of patients with pulmonary tuberculosis. Cytokine. (2023) 171:156374. 10.1016/j.cyto.2023.156374 37782984

[39] Nikitina IY Panteleev AV Kosmiadi GA Serdyuk YV Nenasheva TA Nikolaev AA. Th1, Th17, and Th1Th17 lymphocytes during tuberculosis: Th1 lymphocytes predominate and appear as low-differentiated CXCR3+CCR6+ cells in the blood and highly differentiated CXCR3+/-CCR6- cells in the lungs. J Immunol. (2018) 200:2090–103. 10.4049/jimmunol.1701424 29440351

[40] Kudryavtsev I Zinchenko Y Serebriakova M Akisheva T Rubinstein A Savchenko A. A key role of CD8+ T cells in controlling of tuberculosis infection. Diagnostics. (2023) 13:2961. 10.3390/diagnostics13182961 37761328 PMC10528134

[41] Alduenda JL Choreño-Parra JA Medina-Quero K Zúñiga J Chávez-Galán L. Leukocytes from patients with drug-sensitive and multidrug-resistant tuberculosis exhibit distinctive profiles of chemokine receptor expression and migration capacity. J Immunol Res. (2021) 2021:6654220. 10.1155/2021/6654220 33977111 PMC8084684

[42] Welsh KJ Risin SA Actor JK Hunter RL. Immunopathology of postprimary tuberculosis: increased T-regulatory cells and DEC-205-positive foamy macrophages in cavitary lesions. Clin Dev Immunol. (2011) 2011:307631. 10.1155/2011/307631 21197439 PMC3010642

[43] Hickman HD Reynoso GV Ngudiankama BF Cush SS Gibbs J Bennink JR. CXCR3 chemokine receptor enables local CD8(+) T cell migration for the destruction of virus-infected cells. Immunity. (2015) 42:524–37. 10.1016/j.immuni.2015.02.009 25769612 PMC4365427

[44] Tibúrcio R Narendran G Barreto-Duarte B Queiroz ATL Araújo-Pereira M Anbalagan S. Frequency of CXCR3+ CD8+ T-lymphocyte subsets in peripheral blood is associated with the risk of paradoxical tuberculosis-associated immune reconstitution inflammatory syndrome development in advanced HIV disease. Front Immunol. (2022) 13:873985. 10.3389/fimmu.2022.873985 35432354 PMC9011055

[45] Scriba TJ Kalsdorf B Abrahams DA Isaacs F Hofmeister J Black G. Distinct, specific IL-17- and IL-22-producing CD4+ T cell subsets contribute to the human anti-mycobacterial immune response. J Immunol. (2008) 180:1962–70. 10.4049/jimmunol.180.3.1962 18209095 PMC2219462

[46] Shu CC Wu MF Wang JY Lai HC Lee LN Chiang BL. Decreased T helper 17 cells in tuberculosis is associated with increased percentages of programmed death ligand 1, T helper 2 and regulatory T cells. Respir Res. (2017) 18:128. 10.1186/s12931-017-0580-3 28651576 PMC5485543

[47] Wang T Lv M Qian Q Nie Y Yu L Hou Y. Increased frequencies of T helper type 17 cells in tuberculous pleural effusion. Tuberculosis. (2011) 91:231–7. 10.1016/j.tube.2011.02.002 21371943

[48] Dheda K Chang JS Lala S Huggett JF Zumla A Rook GA. Gene expression of IL17 and IL23 in the lungs of patients with active tuberculosis. Thorax. (2008) 63:566–8. 10.1136/thx.2007.092205 18511642

[49] Chen X Zhang M Liao M Graner MW Wu C Yang Q. Reduced Th17 response in patients with tuberculosis correlates with IL-6R expression on CD4+ T cells. Am J Respir Crit Care Med. (2010) 181:734–42. 10.1164/rccm.200909-1463OC 20019339

[50] Kudryavtsev IV Serebryakova MK Starshinova AA Zinchenko YS Basantsova NY Belyaeva EN. Altered peripheral blood Th17 and follicular T-helper subsets in patients with pulmonary tuberculosis. Russian J Infect Immun. (2019) 9:304–14. 10.15789/2220-7619-2019-2-304-314

[51] Jurado JO Pasquinelli V Alvarez IB Peña D Rovetta AI Tateosian NL. IL-17 and IFN-γ expression in lymphocytes from patients with active tuberculosis correlates with the severity of the disease. J Leukoc Biol. (2012) 91:991–1002. 10.1189/jlb.1211619 22416258 PMC3360475

[52] Marín ND París SC Rojas M García LF. Reduced frequency of memory T cells and increased Th17 responses in patients with active tuberculosis. Clin Vaccine Immunol. (2012) 19:1667–76. 10.1128/CVI.00390-12 22914361 PMC3485891

[53] Vinuesa CG Linterman MA Yu D MacLennan IC. Follicular Helper T Cells. Annu Rev Immunol. (2016) 34:335–68. 10.1146/annurev-immunol-041015-055605 26907215

[54] Morita R Schmitt N Bentebibel SE Ranganathan R Bourdery L Zurawski G. Human blood CXCR5(+)CD4(+) T cells are counterparts of T follicular cells and contain specific subsets that differentially support antibody secretion. Immunity. (2011) 34:108–21. 10.1016/j.immuni.2010.12.012 21215658 PMC3046815

[55] Monin L Griffiths KL Slight S Lin Y Rangel-Moreno J Khader SA. Immune requirements for protective Th17 recall responses to *Mycobacterium tuberculosis* challenge. Mucosal Immunol. (2015) 8:1099–109. 10.1038/mi.2014.136 25627812 PMC4517980

[56] Kahnert A Höpken UE Stein M Bandermann S Lipp M Kaufmann SH. *Mycobacterium tuberculosis* triggers formation of lymphoid structure in murine lungs. J Infect Dis. (2007) 195:46–54. 10.1086/508894 17152008

[57] Phuah JY Mattila JT Lin PL Flynn JL. Activated B cells in the granulomas of nonhuman primates infected with *Mycobacterium tuberculosis*. Am J Pathol. (2012) 181:508–14. 10.1016/j.ajpath.2012.05.009 22721647 PMC3409439

[58] Tsai MC Chakravarty S Zhu G Xu J Tanaka K Koch C. Characterization of the tuberculous granuloma in murine and human lungs: cellular composition and relative tissue oxygen tension. Cell Microbiol. (2006) 8:218–32. 10.1111/j.1462-5822.2005.00612.x 16441433

[59] Flesch IE Kaufmann SH. Activation of tuberculostatic macrophage functions by gamma interferon, interleukin-4, and tumor necrosis factor. Infect Immun. (1990) 58:2675–7. 10.1128/iai.58.8.2675-2677.1990 2115027 PMC258872

[60] Mohan VP Scanga CA Yu K Scott HM Tanaka KE Tsang E. Effects of tumor necrosis factor alpha on host immune response in chronic persistent tuberculosis: possible role for limiting pathology. Infect Immun. (2001) 69:1847–55. 10.1128/IAI.69.3.1847-1855.2001 11179363 PMC98092

[61] Keane J Remold HG Kornfeld H. Virulent *Mycobacterium tuberculosis* strains evade apoptosis of infected alveolar macrophages. J Immunol. (2000) 164:2016–20. 10.4049/jimmunol.164.4.2016 10657653

[62] Algood HM Lin PL Yankura D Jones A Chan J Flynn JLTNF. influences chemokine expression of macrophages *in vitro* and that of CD11b+ cells *in vivo* during *Mycobacterium tuberculosis* infection. J Immunol. (2004) 172:6846–57. 10.4049/jimmunol.172.11.6846 15153503

[63] Shanmuganathan G Orujyan D Narinyan W Poladian N Dhama S Parthasarathy A. Role of interferons in *Mycobacterium tuberculosis* infection. Clin Pract. (2022) 12:788–96. 10.3390/clinpract12050082 36286068 PMC9600403

[64] Herbst S Schaible UE Schneider BE. Interferon gamma activated macrophages kill mycobacteria by nitric oxide induced apoptosis. PLoS ONE. (2011) 6:e19105. 10.1371/journal.pone.0019105 21559306 PMC3085516

[65] Scanga CA Mohan VP Joseph H Yu K Chan J Flynn JL. Reactivation of latent tuberculosis: variations on the Cornell murine model. Infect Immun. (1999) 67:4531–8. 10.1128/IAI.67.9.4531-4538.1999 10456896 PMC96774

[66] Shim TS. Diagnosis and treatment of latent tuberculosis infection due to initiation of anti-TNF therapy. Tuberc Respir Dis. (2014) 76:261–8. 10.4046/trd.2014.76.6.261 25024719 PMC4092157

[67] Ledingham J Wilkinson C Deighton C. British Thoracic Society (BTS) recommendations for assessing risk and managing tuberculosis in patients due to start anti-TNF-{alpha} treatments. Rheumatology. (2005) 44:1205–6. 10.1093/rheumatology/kei103 16172152

[68] Silva DAAD Silva MVD Barros CCO Alexandre PBD Timóteo RP Catarino JS. TNF-α blockade impairs *in vitro* tuberculous granuloma formation and down modulate Th1, Th17 and Treg cytokines. PLoS ONE. (2018) 13:e0194430. 10.1371/journal.pone.0194430 29543912 PMC5854376

[69] van Pinxteren LA Cassidy JP Smedegaard BH Agger EM Andersen P. Control of latent *Mycobacterium tuberculosis* infection is dependent on CD8 T cells. Eur J Immunol. (2000) 30:3689–98. 10.1002/1521-4141(200012)30:12&lt;3689::AID-IMMU3689&gt;3.3.CO;2-W 11169412

[70] Gao XF Yang ZW Li J. Adjunctive therapy with interferon-gamma for the treatment of pulmonary tuberculosis: a systematic review. Int J Infect Dis. (2011) 15:e594–600. 10.1016/j.ijid.2011.05.002 21715206

[71] Foreman TW Mehra S LoBato DN Malek A Alvarez X Golden NA. CD4+ T-cell-independent mechanisms suppress reactivation of latent tuberculosis in a macaque model of HIV coinfection. Proc Natl Acad Sci USA. (2016) 113:E5636–44. 10.1073/pnas.1611987113 27601645 PMC5035858

[72] Selwyn PA Hartel D Lewis VA Schoenbaum EE Vermund SH Klein RS. A prospective study of the risk of tuberculosis among intravenous drug users with human immunodeficiency virus infection. N Engl J Med. (1989) 320:545–50. 10.1056/NEJM198903023200901 2915665

[73] Berbudi A Rahmadika N Tjahjadi AI Ruslami R. Type 2 diabetes and its impact on the immune system. Curr Diabetes Rev. (2020) 16:442–9. 10.2174/1573399815666191024085838 31657690 PMC7475801

[74] Jeon CY Murray MB. Diabetes mellitus increases the risk of active tuberculosis: a systematic review of 13 observational studies. PLoS Med. (2008) 5:e152. 10.1371/journal.pmed.0050152 18630984 PMC2459204

[75] Fortes FML Rocha R Santana GO. Thiopurines are an independent risk factor for active tuberculosis in inflammatory bowel disease patients. World J Gastroenterol. (2023) 29:1536–8. 10.3748/wjg.v29.i9.1536 36998430 PMC10044854

[76] Wu PH Lin YT Yang YH Lin YC Lin YC. The increased risk of active tuberculosis disease in patients with dermatomyositis – a nationwide retrospective cohort study. Sci Rep. (2015) 5:16303. 10.1038/srep16303 26573418 PMC4647179

[77] Aguado JM Herrero JA Gavaldá J Torre-Cisneros J Blanes M Rufí G. Clinical presentation and outcome of tuberculosis in kidney, liver, and heart transplant recipients in Spain. Spanish Transplantation Infection Study Group, GESITRA *Transplantation*. (1997) 63:1278–86. [published correction appears in Transplantation 64, 942]. 10.1097/00007890-199705150-00015 9158022

[78] Steiger S Rossaint J Zarbock A Anders HJ. Secondary Immunodeficiency related to kidney disease (SIDKD)-definition, unmet need, and mechanisms. J Am Soc Nephrol. (2022) 33:259–78. 10.1681/ASN.2021091257 34907031 PMC8819985

[79] Stachowski J Pollok M Burrichter H Spithaler C Baldamus CA. Signaling via the TCR/CD3 antigen receptor complex in uremia is limited by the receptors number. Nephron. (1993) 64:369–75. 10.1159/000187356 8101978

[80] Moser B Roth G Brunner M Lilaj T Deicher R Wolner E. Aberrant T cell activation and heightened apoptotic turnover in end-stage renal failure patients: a comparative evaluation between non-dialysis, haemodialysis, and peritoneal dialysis. Biochem Biophys Res Commun. (2003) 308:581–5. 10.1016/S0006-291X(03)01389-5 12914790

[81] Hussein MM Mooij JM Roujouleh H. Tuberculosis and chronic renal disease. Semin Dial. (2003) 16:38–44. 10.1046/j.1525-139X.2003.03010.x 12535299

[82] Andrew OT Schoenfeld PY Hopewell PC Humphreys MH. Tuberculosis in patients with end-stage renal disease. Am J Med. (1980) 68:59–65. 10.1016/0002-9343(80)90166-7 7350806

[83] Belcon MC Smith EK Kahana LM Shimizu AG. Tuberculosis in dialysis patients. Clin Nephrol. (1982) 17:14–8. 7055993

[84] Shovkun LA Aksenova VA Kudlay DA Sarychev AM. The role of immunological tests in the diagnosis of tuberculosis infection in children with juvenile idiopathic arthritis. Eur Respir J Suppl. (2018) 52:A2733. 10.1183/13993003.congress-2018.PA2733

[85] Starshinova A Osipov N Dovgalyk I Kulpina A Belyaeva E Kudlay D. COVID-19 and tuberculosis: mathematical modeling of infection spread taking into account reduced screening. Diagnostics. (2024) 14:698. 10.3390/diagnostics14070698 38611611 PMC11011507

[86] Jutel M Agache I Zemelka-Wiacek M Akdis M Chivato T Del Giacco S. Nomenclature of allergic diseases and hypersensitivity reactions: adapted to modern needs: an EAACI position paper. Allergy. (2023) 78:2851−74. 10.1111/all.15889 37814905

[87] Swanson RV Gupta A Foreman TW Lu L Choreno-Parra JA Mbandi SK. Antigen-specific B cells direct T follicular-like helper cells into lymphoid follicles to mediate *Mycobacterium tuberculosis* control. Nat Immunol. (2023) 24:855–68. 10.1038/s41590-023-01476-3 37012543 PMC11133959

[88] Crotty S. T follicular helper cell biology: a decade of discovery and diseases. Immunity. (2019) 50:1132–48. 10.1016/j.immuni.2019.04.011 31117010 PMC6532429

